# Supplementary material for: Splice-Junction-Based Mapping of Alternative Isoforms in the Human Proteome
Source: Cell Rep. Author manuscript; Available in PMC 2020 Jan 15. (PMC6961840; doi:10.1016/j.celrep.2019.11.026)
Supplement: 2 [file NIHMS1546469-supplement-2.zip › DF1/PXD006675/PulmonaryArtery/PulmonaryArtery_22_RFWD2_QLEAQLEQIQK.pdf]

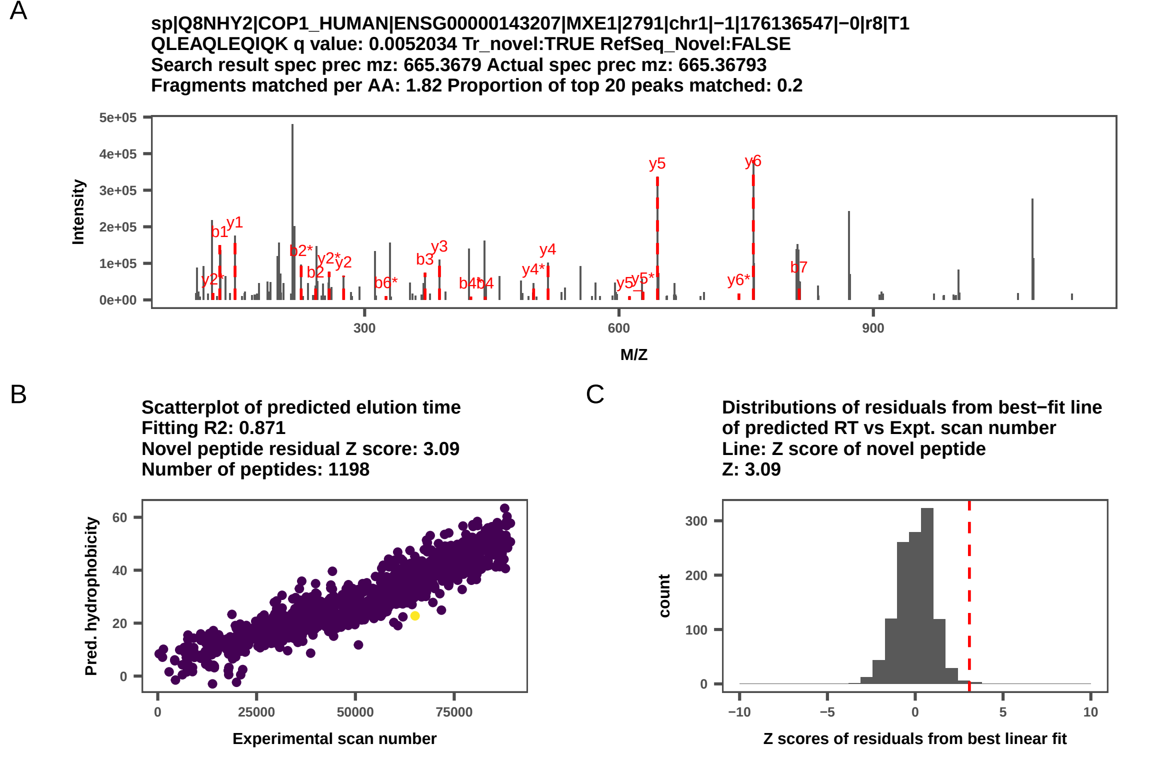

A
sp|Q8NHY2|COP1_HUMAN|ENSG00000143207|MXE1|2791|chr1|−1|176136547|−0|r8|T1
QLEAQLEQIQK q value: 0.0052034 Tr_novel:TRUE RefSeq_Novel:FALSE
Search result spec prec mz: 665.3679 Actual spec prec mz: 665.36793
Fragments matched per AA: 1.82 Proportion of top 20 peaks matched: 0.2
Intensity
M/Z
B
Scatterplot of predicted elution time
Fitting R2: 0.871
Novel peptide residual Z score: 3.09
Number of peptides: 1198
Pred. hydrophobicity
Experimental scan number
C
Distributions of residuals from best−fit line
of predicted RT vs Expt. scan number
Line: Z score of novel peptide
Z: 3.09
count
Z scores of residuals from best linear fit
